# Re-evaluating transcranial static magnetic stimulation (tSMS): No inhibitory effects on motor cortex across hemispheres

**DOI:** 10.1016/j.cnp.2026.02.008

**Published:** 2026-03-04

**Authors:** Sabrina Lorenz, Linda Bartusch, Cornelia Heusler, Stefan Götz, Thomas Kammer

**Affiliations:** a Section for Neurostimulation, Department of Psychiatry, University of Ulm, Ulm, Germany; b Department of Electrical and Computer Engineering, RPTU Kaiserslautern, Kaiserslautern, Germany; c Neuroscience Center Ulm, University of Ulm, Ulm, Germany

**Keywords:** tSMS, Static magnetic field, MEP variability, Motor system, Replication study, SICI, ICF

## Abstract

**Objective:**

To investigate whether transcranial static magnetic stimulation (tSMS) exerts inhibitory effects on the human motor cortex and whether these effects differ between dominant and non-dominant hemispheres.

**Methods:**

Participants underwent tSMS (20 min) and sham stimulation, targeting either the left (dominant, n = 19) or right (non-dominant, n = 19) motor cortex in a between-subject design. Corticospinal excitability was assessed with transcranial magnetic stimulation (TMS), using single-pulse motor evoked potentials (MEPs) and paired-pulse paradigms (short-interval intracortical inhibition SICI, intracortical facilitation ICF). Neuronavigation ensured stable hotspot localization.

**Results:**

No significant differences in MEP amplitudes were observed between tSMS and sham stimulation. Paired-pulse measures confirmed robust inhibition and facilitation, with higher reliability for SICI. However, neither SICI nor ICF showed modulation by tSMS.

**Conclusions:**

tSMS modulated excitability in neither the dominant nor the non– dominant hemisphere. The results suggest that previously reported effects may reflect methodological limitations, particularly absence of neuronavigation or low trial numbers.

**Significance:**

This study provides the first direct hemispheric comparison of tSMS using neuronavigation and robust stimulation protocols, questioning the robustness of earlier findings and highlighting methodological standards for future research.

## Highlights

• Transcranial static magnetic stimulation did not alter motor-evoked potentials.
• tSMS did not affect short-interval intracortical inhibition or facilitation.
• intracortical inhibition showed higher reliability than intracortical facilitation.

## 1 Introduction

In the initial description of transcranial static magnetic field stimulation (tSMS) in 2011 (Oliviero et al., 2011), it was demonstrated that, compared to a sham magnet, a 10-minute application of tSMS produced an inhibitory effect on motor cortex excitability that persisted for up to six minutes after stimulation. This inhibitory effect has since been replicated in several studies (Arias et al., 2017, Dileone et al., 2018, Kirimoto et al., 2016, Lacroix et al., 2019, Nojima et al., 2015, Shibata et al., 2020, Silbert et al., 2013, Takamatsu et al., 2021). However, some exceptions exist – most notably the study by Kufner and colleagues (Kufner et al., 2017), in which no such inhibitory effect could be reproduced, despite a thorough methodological review that revealed no clear explanation.

In this particular study, participants performed an auditory Oddball task during the tSMS intervention, wherein tones were presented in random sequence and the rarest tones had to be counted to ensure attentional engagement. One proposed explanation for the absence of the expected tSMS effect was that this Oddball task might have acted as a distraction. Additionally, the act of counting may have activated the motor cortex, potentially masking any inhibitory effects (Foffani and Dileone, 2017). However, this hypothesis was later refuted in a follow-up study, where participants received 10-minute tSMS sessions both with and without the Oddball task – and in both cases, no inhibition of motor cortex excitability was observed (Lorenz et al., 2020).

Other research groups have also failed to replicate the inhibitory effects of tSMS. For example, Hamel et al. (Hamel et al., 2020) were unable to confirm the findings of an earlier study that reported reduced MEP amplitudes for 30 min following a 30-minute tSMS session (Dileone et al., 2018). Hamel and colleagues proposed that the limited sample size (n = 10) in the original study may have led to an overestimation of their effect sizes. Similarly, a study in children found no effects of tSMS on MEP amplitudes, short-interval intracortical inhibition (SICI), or intracortical facilitation (ICF), although behavioral effects on motor learning were observed (Hollis et al., 2020).

One potential explanation for these inconsistencies lies in methodological variability. Structural differences in the motor cortex between left- and right-handed individuals, such as an increased cortical volume in the dominant hemisphere (Amunts et al., 1996), were frequently overlooked, as many studies did not report participants’ handedness. Furthermore, there is little uniformity in the selection of the hemisphere for stimulation. Some studies applied tSMS to the right hemisphere and measured motor evoked potentials (MEPs) from the contralateral (left) hand, without accounting for handedness (Arias et al., 2017, Oliviero et al., 2011). Others targeted the left hemisphere and assessed muscle activity in the right hand, yet similarly failed to document handedness (Hamel et al., 2020, Silbert et al., 2013). Even among studies that did report handedness, stimulation was inconsistently applied – sometimes to the left hemisphere (Kirimoto et al., 2016, Kufner et al., 2017, Lorenz et al., 2020, Nojima et al., 2015, Takamatsu et al., 2021), and at other times to the right (Dileone et al., 2018, Shibata et al., 2020). As a result, it remains unclear whether the dominant or non-dominant hemisphere was stimulated, further complicating the interpretation of findings across studies.

Only a limited number of studies have utilized neuronavigation to guide coil placement (Davila-Pérez et al., 2019, Hamel et al., 2020, Hollis et al., 2020, Kufner et al., 2017, Lorenz et al., 2020), and notably, the majority of these did not observe significant tSMS effects. Neuronavigation offers improved precision in coil positioning and ensures consistency across sessions, particularly in real-sham within-subject designs.

The present study set out to further explore potential methodological factors contributing to the inconsistent replication of tSMS-induced inhibitory effects. To enhance statistical power, the sample size was increased. In addition, tSMS was applied to both hemispheres using a between-group design and limited to right-handed participants, allowing for a direct comparison between the dominant and non-dominant hemispheres. Coil placement was once again guided by neuronavigation to ensure spatial accuracy. Finally, the effects of tSMS were assessed not only on single-pulse MEPs, but also on paired-pulse paradigms such as short-interval intracortical inhibition (SICI) and intracortical facilitation (ICF), which offer deeper insights into the excitability of intracortical inhibitory and excitatory networks.

## 2 Methods

### 2.1 Subjects

For this study, 39 right-handed subjects (accessed using a modified version of the Edinburgh Handedness Inventory (Oldfield, 1971) were recruited. Two of them had to be excluded because, despite of high TMS intensities, no MEP amplitudes of 1 mV could be elicited. Of the remaining 37 subjects (15 men, mean age 25.31 ± 3.52 years), five participated with both hemispheres, resulting in 42 evaluable measurements (21 per hemisphere).

All subjects were free of neurological and psychiatric disorders, chronic diseases, metal implants – particularly in the head region – any history of former brain surgery, and substance abuse. Participants were instructed to avoid alcohol on the preceding evening, ensure adequate sleep, and refrain from consuming coffee on the day of testing. Other than oral contraceptives, no medications were taken. All gave their written informed consent and received financial compensation. The study was approved by the local ethics committee (357/19) and conducted in accordance with the Declaration of Helsinki.

### 2.2 Measurement of motor cortical excitability

First, the motor hotspot of the respective hemisphere was located using a MagPro X100 stimulator (MagVenture A/S, Farum, Denmark) connected to a figure-of-eight-coil (MC-B70, MagVenture).

MEPs of the contralateral first dorsal interosseus (FDI) muscle at rest were evoked by monophasic single TMS pulses, with the coil held tangentially to the scalp and the handle pointing backward at a 45° angle to the sagittal plane. The site producing the largest MEPs was defined as the motor hotspot and marked on the participant’s scalp. Coil position was also stored using a neuronavigation system (PowerMAG View!, MAG & More, Munich, Germany) to ensure consistent positioning throughout the session and across sessions.

Signals were amplified using a Toennies Universal Amplifier (Erich Jaeger GmbH, Höchberg, Germany) with a bandpass filter of 10–2000 Hz, and sampled at 5000 Hz. MEPs were recorded and displayed using DasyLab 13.0 (measX GmbH & Co. KG, Mönchengladbach, Germany), and stored for offline analysis. For each pulse, 800 ms of data before and after stimulation were recorded. Muscle innervation of the FDI was continuously monitored both visually and acoustically to detect pre-innervation and ensure muscle relaxation.

The resting motor threshold (RMT) was defined as the lowest TMS intensity that evoked MEPs of approximately 50 μV in at least 5 out of 10 trials. This threshold was determined at the beginning of each session, as was the 1 mV intensity, the TMS intensity that evoked MEPs averaging approximately 1 mV (range: 0.8–1.3 mV) over 10 single pulses. Both RMT and 1 mV intensity were determined using a manual Rossini–Rothwell titration procedure, adjusting stimulus intensity in small steps (2% or 1% of maximum stimulator output, MSO) until the respective criterion was met.

### 2.3 Study design

Baseline excitability was assessed in two pre-measurements (pre 1 and pre 2), each consisting of 60 pulses. These were administered alternately using monophasic single pulses (1 mV intensity) and two paired-pulse protocols: SICI (interstimulus interval, ISI = 2 ms) and ICF (ISI = 12 ms). Each pre-measurement included 20 test pulses, 20 SICI, and 20 ICF stimuli. Stimulation intensities were set at 80% RMT for the subthreshold conditioning stimulus and 1 mV intensity for the suprathreshold test stimulus. Stimuli were applied in a fixed sequence with one control stimulus followed by a SICI and an ICF stimulus. The frequency of stimulation was randomized between 0.125 Hz and 0.2 Hz (i.e., one pulse every 5–8 s). Each block took approximately 7–8 min. To standardize the timing, the second baseline block began exactly 15 min after the first.

Subsequently, either tSMS or sham stimulation (within-subject design) was applied for 20 min over the motor hotspot of either the left or right hemisphere (between-subject design). Post-intervention measurements (post 1 and post 2) were identical in structure to the pre- measurements and began exactly one minute after the intervention ended. The whole session lasted about 90–120 min.

**Fig. 1:**
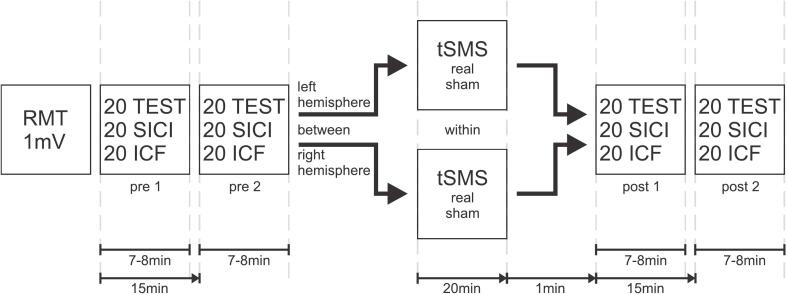
Schematic representation of the study design. Each session included RMT and 1 mV determination, two pre-intervention measurements (7–8 min each, 15 min apart), a 20-min tSMS or sham intervention (within-subject), and post-intervention measurements starting 1 min after stimulation. Hemispheres were tested between subjects. MEP = motor-evoked potential, tSMS = transcranial static magnetic field stimulation.

Participants returned for their second session after an average interval of 26.58 days (SD 40.68 days, range 2 – 184 days). The order of interventions (tSMS vs. sham) was counterbalanced across participants. Both sessions were scheduled at approximately the same time of day (± 30 min) to minimize circadian variability. All measurements were conducted by the same two trained operators, to ensure consistent hotspot identification and handling procedures. A schematic representation of the study design is shown in Fig. 1.

### 2.4 Transcranial static magnetic field stimulation (tSMS)

tSMS was administered using an axially magnetized cylindrical N45 neodymium-iron-boron magnet (model S-45–30-N, Webcraft GmbH, Gottmadingen, Germany), 30 mm in height and 45 mm in diameter, with a holding force of approximately 677 N (69 kg). Since the polarity of the magnet has been shown not to affect the outcome of tSMS (Oliviero et al., 2011), the south pole was consistently placed over the identified motor hotspot and held in position for 20 min. The hotspot had previously been marked with a skin-friendly marker.

In the sham condition, a stainless-steel cylinder identical in size, weight, and appearance to the real magnet was used. To ensure a double-blind design, both real and sham magnets were handed over by a third person, so that neither the participants nor the experimenters knew which was applied.

### 2.5 Data analysis

Data were analyzed using Statistica (v13, StatSoft GmbH, Hamburg, Germany).

MEP data were preprocessed using Matlab (version 9.7.0.1216025 (R2019b), The MathWorks, Natick, MA, USA). Each dataset was checked for pre-innervation (within 200 ms before the pulse) and silent periods (inhibitory responses up to 500 ms post-stimulation). Datasets were excluded if more than 7% of trials were affected (n = 4). Thus, 19 datasets per hemisphere were included in the final analysis. Data from participants who were measured on both hemispheres were treated as between-subject data.

MEP amplitudes were averaged across measurement points (pre 1, 2; post 1, 2), session (real vs. sham), and stimulation type (test pulse, SICI, ICF). Imputations were performed at eight measurement time points due to pre-innervation, which necessitated the exclusion of some data points. Missing values were replaced by the group mean of the corresponding time point within the same hemisphere group. No imputation was performed across hemispheres, interventions, or time points.

Data were analyzed using repeated measures analyses of variance (rmANOVA). Mauchly’s test was used to check for sphericity; when violated, Greenhouse-Geisser corrections were applied. Corrected degrees of freedom, p-values, and ε are reported where applicable. For the primary analysis, we used absolute (unnormalized) MEP amplitudes because ratio-based normalization can inflate variability and produce extreme values when baseline test MEPs are small. This approach provided more stable estimates of corticospinal and intracortical responses.

In response to reviewer feedback and to ensure comparability with prior TMS literature, we additionally performed a supplementary analysis using normalized data (single-pulse MEPs relative to the pre mean; SICI/ICF as conditioned/test ratios). These results are provided in the supplementary material and lead to the same overall conclusions.

Lastly, correlation coefficients were calculated to assess the relationships between RMT and 1 mV intensities, as well as between RMT and participant age. These were tested for statistical significance.

## 3 Results

### 3.1 Threshold values

To first assess the reliability of the threshold values, the resting motor threshold (RMT) and the 1 mV intensity measured at the beginning of each of the two sessions were statistically analyzed using repeated-measures ANOVAs, with SESSION (1, 2) as a within-subject factor and HEMISPHERE (left, right) as a between-subjects factor.

Numerical differences between hemispheres were observed, with higher values for the right hemisphere (left: RMT = 40.7% MSO, 1 mV intensity = 51.7% MSO; right: RMT = 43.3% MSO, 1 mV intensity = 57.5% MSO). However, these differences were not statistically significant (main effect HEMISPHERE RMT: F_(1,36)_ = 1.32, p = 0.26, η^2^ = 0.04; 1 mV intensity: F_(1,36)_ = 3.48, p = 0.07, η^2^ = 0.09). No significant main effects for SESSION or interactions were found (all p > 0.30).

These results suggest that both RMT and 1 mV intensities were stable across sessions. Furthermore, RMT and 1 mV values were strongly correlated within each hemisphere (left hemisphere session 1: r = 0.92, p < 0.001; session 2: r = 0.93, p < 0.001; right hemisphere session 1: r = 0.94, p < 0.001; session 2: r = 0.80, p < 0.001).

There was no significant correlation between participants' age and either RMT (left hemisphere: r = -0.10, p = 0.68; right hemisphere: r = 0.20, p = 0.41) or the 1 mV intensity (left: r = -0.16, p = 0.52; right: r = 0.26, p = 0.27) as analyzed using the values of the first session.

### 3.2 tSMS effects on MEP amplitudes

To examine potential effects of tSMS on the motor cortex and to assess hemispheric lateralization, a repeated-measures ANOVA was conducted on the MEP amplitudes elicited by 1 mV control pulses. The within-subject factors were INTERVENTION (tSMS, sham) and TIME (pre 1, pre 2, post 1, post 2), and the between-subject factor was HEMISPHERE (left, right). The average MEP amplitudes at pre 1 and pre 2 reflect the excitability level prior to the intervention, while post 1 and post 2 indicate the excitability level afterward.

**Fig. 2:**
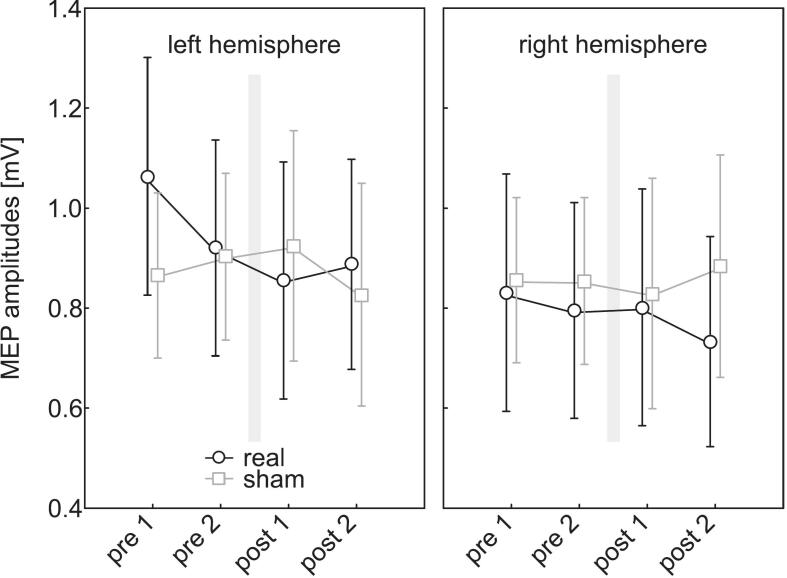
Effect of tSMS on MEP amplitudes. Shown are the mean MEP amplitudes for single pulses at 1 mV intensity at the different time points (pre 1, pre 2, post 1, post 2). Left panel: participants receiving stimulation over the left (dominant) hemisphere; right panel: participants receiving stimulation over the right (non-dominant) hemisphere. The intervention (20 min, grey bars) took place between pre 2 and post 1. Black = real tSMS, grey = sham stimulation. Error bars represent 95% confidence intervals. MEP = motor-evoked potential, tSMS = transcranial static magnetic field stimulation.

Neither the main effect of INTERVENTION (F_(1,36)_ = 0.01, p = 0.93, η^2^ < 0.01) nor that of TIME (F_(3, 108)_ = 0.97, p = 0.41, η^2^ = 0.03) or HEMISPHERE (F_(1,36)_ = 0.63, p = 0.43, η^2^ = 0.02) reached statistical significance (see Fig. 2).

All interaction effects were also nonsignificant (all p > 0.27).

An additional analysis using normalized data is provided in supplement S1.

**Fig. 3:**
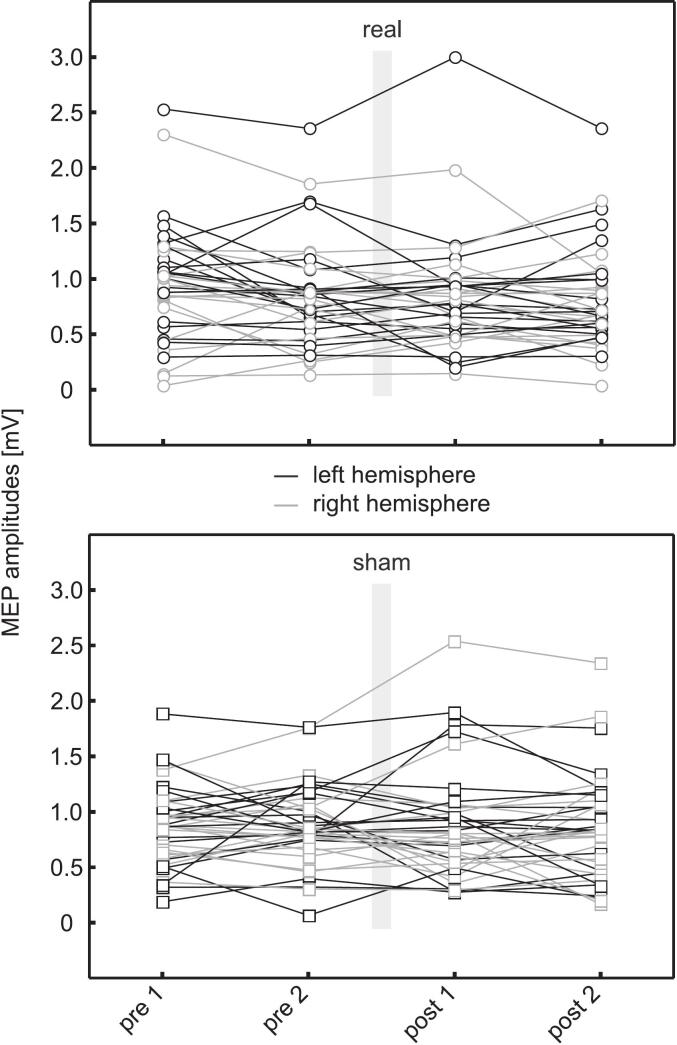
Individual MEP amplitudes during real and sham tSMS sessions. Shown are the mean MEP amplitudes for single pulses at 1 mV intensity at the different time points (pre 1, pre 2, post 1, post 2) for each participant. Upper panel: real tSMS sessions; lower panel: sham tSMS sessions. Black = left (dominant) hemisphere, grey = right (non-dominant) hemisphere. The intervention (20 min, grey bars) took place between pre 2 and post 1. MEP = motor-evoked potential, tSMS = transcranial static magnetic field stimulation.

To better illustrate the interindividual variability in MEP amplitudes, individual participant data are presented in Fig. 3.

### 3.3 Paired-pulse paradigms SICI/ICF

The reliability of the measured parameters was first assessed using correlation analyses. To avoid potential influence from the tSMS intervention, only the four *pre* values per participant were used to determine the intra- and inter-session reliability of the parameters test MEP, SICI, and ICF. As no hemispheric differences were observed in these analyses, results for both hemispheres are reported together for clarity. SICI and ICF MEP amplitudes normalized to the respective test pulse are not presented, as normalization did not alter the results and would only provide a distorted representation of the measurements. Normalization often produces extreme ICF values in cases where statistical outliers occur in the test pulse data, which disproportionately increases standard deviations without improving the interpretability of the results. An additional analysis using normalized data is provided in supplement S2.

The *r*- and corresponding *p*-values from the correlation analyses are presented in Table 1.

**Table 1:** Correlation analyses.

		Testpuls	SICI	ICF
**intra- session**	s1 pre1/s1 pre2	r = 0.71 p < 0.01	r = 0.79 p < 0.01	r = 0.81 p < 0.01
	s2 pre1/s2 pre2	r = 0.77 p < 0.01	r = 0.84 p < 0.01	r = 0.75 p < 0.01
**inter- session**	s1 pre1/s2 pre1	r = 0.28 p = 0.09	r = 0.41 p = 0.01	r = 0.32 p = 0.05
	s1 pre1/s2 pre2	r = 0.25 p = 0.13	r = 0.52 p < 0.01	r = 0.16 p = 0.32
	s1 pre2/s2 pre1	r = 0.33 p = 0.04	r = 0.41 p = 0.01	r = 0.22 p = 0.18
	s1 pre2/s2 pre2	r = 0.27 p = 0.11	r = 0.50 p < 0.01	r = 0.15 p = 0.37

Shown are the *r*- and *p*-values for the correlations of the mean MEP amplitudes for the parameters test pulse, SICI, and ICF across the four measurement time points: s1 pre1 (session 1, measurement 1), s1 pre2 (session 1, measurement 2), s2 pre1 (session 2, measurement 1), and s2 pre2 (session 2, measurement 2).

The effects of tSMS on the paired-pulse stimulation patterns SICI and ICF were analyzed separately for each hemisphere.

Left hemisphere

The recorded MEP amplitudes were analyzed using a three-factor rmANOVA with the within-subject factors PULSE (test, SICI, ICF), INTERVENTION (real, sham), and TIME (pre 1, pre 2, post 1, post 2).

Mauchly’s test indicated a violation of sphericity for the factor PULSE, and Greenhouse-Geisser correction was applied. A statistically significant main effect was found for PULSE (F_(1.53,27.47)_ = 74.27, p ≤ 0.001, ε = 0.76). Post hoc Bonferroni tests revealed significant inhibition for SICI (p < 0.001) and significant facilitation for ICF (p = 0.011) compared to the test pulse.

**Fig. 4:**
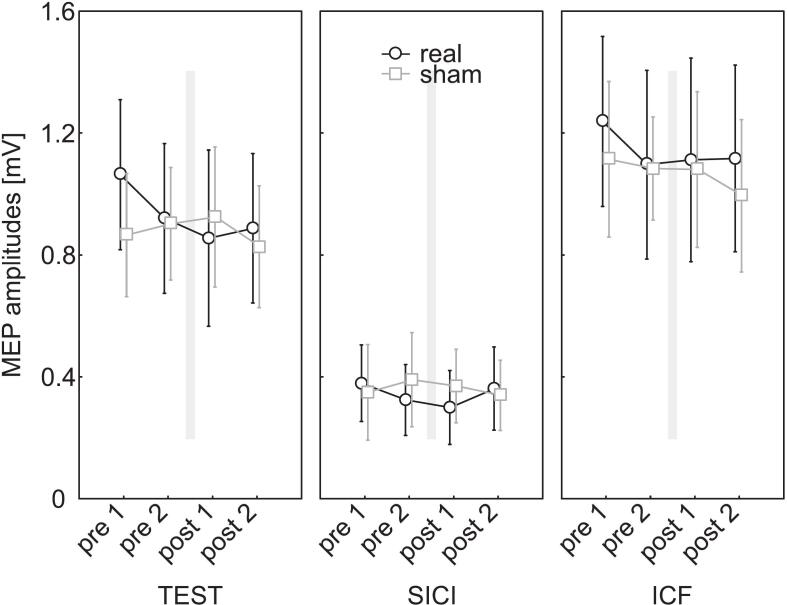
Effect of tSMS on SICI and ICF in the left (dominant) hemisphere. Shown are the mean MEP amplitudes for the test pulse and the paired-pulse paradigms at the different time points (pre 1, pre 2, post 1, post 2) during real tSMS (black) and sham (grey) stimulation. The intervention (20 min, grey bar) took place between pre 2 and post 1. Error bars represent 95% confidence intervals. MEP = motor-evoked potential; tSMS = transcranial static magnetic field stimulation; SICI = short-interval cortical inhibition; ICF = intracortical facilitation.

The main effects of INTERVENTION (F_(1,18)_ = 0.14, p = 0.71, η^2^ = 0.01) and TIME (F_(3,54)_ = 0.82, p = 0.49, η^2^ = 0.04) were not statistically significant, nor were any of the possible interactions (all p > 0.23). The results are illustrated in Fig. 4.

Right Hemisphere

As for the left hemisphere, the recorded MEP amplitudes were analyzed using a three-way repeated-measures ANOVA with the within-subject factors PULSE (test, SICI, ICF), INTERVENTION (real, sham), and TIME (pre 1, pre 2, post 1, post 2).

Mauchly’s test indicated a violation of sphericity for the factor PULSE, as well as for TIME and the PULSE*TIME interaction; Greenhouse-Geisser correction was applied accordingly. A significant main effect was found for PULSE (F_(1.34,24.04)_ = 126.96, p < 0.001, ε = 0.67). Post hoc Bonferroni tests revealed significant inhibition for SICI (p < 0.001) and significant facilitation for ICF (p < 0.001) compared to the test pulse.

The main factors INTERVENTION (F_(1,18)_ = 0.58, p = 0.46, η^2^ = 0.03) and TIME (F_(1.79,32.3)_ = 0.26, p = 0.75, ε = 0.60) were not statistically significant. None of the possible interactions reached significance (all p > 0.28), except for the Mauchly-violating PULSE × TIME interaction. However, this effect was no longer significant after Greenhouse–Geisser correction (F_(3.94,70.96)_ = 2.27, p < 0.07, ε = 0.66).

**Fig. 5:**
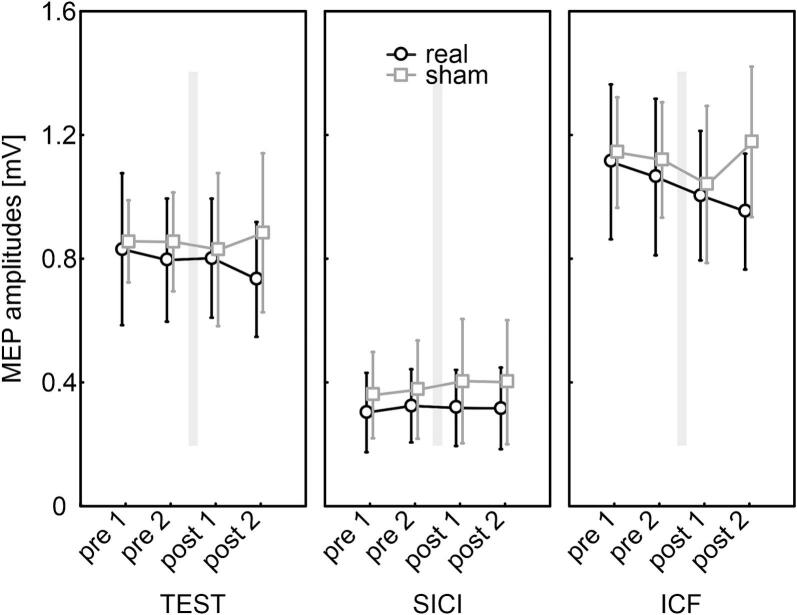
Effect of tSMS on SICI and ICF of the right (non-dominant) hemisphere. Shown are the mean MEP amplitudes for the test pulse and the paired-pulse paradigms at the different time points (pre 1, pre 2, post 1, post 2) during real tSMS (black) and sham (grey) stimulation. The intervention (20 min, grey bar) took place between pre 2 and post 1. Error bars represent 95% confidence intervals. MEP = motor-evoked potential, tSMS = transcranial static magnetic field stimulation, SICI = short-interval cortical inhibition, ICF = intracortical facilitation.

The results are illustrated in Fig. 5.

Additionally, to isolate corticospinal excitability from intracortical inhibition/facilitation effects and to better examine potential hemisphere-specific effects, we analyzed the SICI and ICF data separately.

For SICI, we conducted an ANOVA with the within-subject factors INTERVENTION (real, sham) and TIME (pre 1, pre 2, post 1, post 2) as well as the between-subject factor HEMISPHERE (left, right). None of the main effects (INTERVENTION: F_(1,36)_ = 0.96, p = 0.33, η^2^ = 0.03; TIME: F_(3,108)_ = 0.05, p = 0.98, η^2^ < 0.01; HEMISPHERE: F_(1,36)_ < 0.01, p = 0.98, η^2^ < 0.01) or interactions (all p > 0.38) reached statistical significance (see supplementary Fig. S2).

Similarly, for ICF we conducted an ANOVA with the within-subject factors INTERVENTION (real, sham) and TIME (pre 1, pre 2, post 1, post 2) and the between-subject factor HEMISPHERE (left, right). Again, none of the main effects (INTERVENTION: F_(1,36)_ < 0.01, p = 0.94, η^2^ < 0.01; TIME: F_(3,108)_ = 1.80, p = 0.15, η^2^ = 0.05; HEMISPHERE: F_(1,36)_ = 0.06, p = 0.81, η^2^ < 0.01) or interactions (all p > 0.27) reached statistical significance (see supplementary Fig. S2).

Further analyses using normalized data is provided in supplement S2.

## 4 Discussion

The aim of the present study was to investigate the influence of a static magnetic field on the excitability of the human motor cortex, with particular focus on replicating previous studies that reported inhibitory effects of tSMS following motor cortex stimulation (e.g. Dileone et al., 2018, Oliviero et al., 2011, Silbert et al., 2013). We also aimed to examine potential hemisphere-specific effects, motivated by the hypothesis that inconsistent reporting of the stimulated hemisphere in earlier studies might have contributed to unsuccessful replication attempts in our own prior work (Kufner et al., 2017, Lorenz et al., 2020). Beyond single-pulse MEPs, we assessed paired-pulse paradigms – short-interval intracortical inhibition (SICI) and intracortical facilitation (ICF) – to provide a more comprehensive view of cortical excitability changes. We did not find any tSMS modulation on motor cortex excitability, neither in MEPs nor in the two paired-pulse paradigms.

Although the right hemisphere exhibited numerically higher threshold values than the left, differences were not statistically significant. While the two hemispheres were tested in a between-subject design in the present study, other studies have also reported non-significant differences in RMT between hemispheres using a within-subject design (Civardi et al., 2000, Cueva et al., 2016, Maeda et al., 2002, Souza et al., 2018). In contrast, these hemisphere-specific differences in RMT reached statistical significance in other studies (Macdonell et al., 1991, Matsunaga et al., 1998, Triggs et al., 1994). Both RMT and 1 mV intensities in our study showed good reliability across measurement days for each hemisphere, consistent with previous findings (Brown et al., 2017, Lorenz et al., 2020, Maeda et al., 2002, Sale et al., 2007).

Paired-pulse paradigms produced the expected significant inhibition and facilitation for SICI and ICF, respectively. Stronger reliabilities were observed for SICI than for ICF across the two sessions. This observation is in line with reports by Maeda et al. (2002), who also demonstrated a stronger correlation for intracortical inhibition compared to intracortical facilitation between two sessions. Biabani et al. (2018) likewise reported better inter-session reliability for SICI than for ICF. This may be explained by the involvement of different signaling pathways. While SICI mainly relies on a GABAergic pathway, ICF involves two receptor types, GABA and N-methyl-D-aspartate (NMDA). This dual involvement may account for reduced replicability (Biabani et al., 2018).

In the present study, no inhibitory effect of tSMS on the motor cortex was observed. MEP amplitudes did not differ between real and sham tSMS for either hemisphere, which indicates that the choice of hemisphere cannot explain the absence of tSMS effects in previous studies from our group (Kufner et al., 2017, Lorenz et al., 2020). To our knowledge, only three studies have examined TMS measures after tSMS in both hemispheres (Hollis et al., 2020, Pagge et al., 2025, Takamatsu et al., 2021). Hollis et al., (2020) stimulated both hemispheres but measured excitability only in the right, while Takamatsu (2021) stimulated the dominant hemisphere and reported decreased ipsilateral and increased contralateral excitability.

Only in one recent study (Pagge et al., 2025), the effect of tSMS on motor cortex excitability of both hemispheres was examined. In this study, unlike in our results, an inhibitory effect of tSMS on the motor cortex was reported, which was specifically observed following stimulation of the right hemisphere. The authors revealed anecdotal-to-moderate evidence for a larger reduction of corticospinal excitability with right M1 compared to left M1 tSMS. However, no tSMS effect was observed in either hemisphere in our study.

The main differences between the two studies include the use of a different magnet and other methodological variations in measuring cortical excitability. For example, in our study, two pre-tSMS measurements were conducted, allowing us to capture the intrinsic variability of the measures even in the absence of tSMS effects. Inspection of the individual data in Fig. 3 shows that the values of the two pre-measurements within a participant often fluctuate as much as the values between pre- and post-measurements. This baseline variability is neglected when only a single pre-measurement is taken and especially when a sham condition is omitted, and may have contributed to the discrepancy between our results and those of Pagge et al. (2025). It is also very likely that the use of a considerably stronger magnet and a longer stimulation time in their study (30 min vs. 20 min in our study) accounted for the pronounced inhibition observed. This is further illustrated by their third experiment, where a smaller magnet produced weaker effects. In any case, we used the same magnet as in the first tSMS studies (Oliviero et al., 2011, Silbert et al., 2013) and observed no effects in either hemisphere. Compared to the other studies, it is therefore not possible to conclusively determine whether the choice of stimulated hemisphere explains the absence of tSMS effects in our research group. Further investigations, for example involving left-handed individuals, are necessary in order to clarify this question in more detail.

A closer look at the literature suggests a correlation between the absence of tSMS effects and the use of neuronavigation. Studies reporting tSMS effects typically did not use neuronavigation (Arias et al., 2017, Dileone et al., 2018, Nojima et al., 2015, Oliviero et al., 2011, Silbert et al., 2013, Takamatsu et al., 2021), whereas most neuronavigation studies failed to demonstrate effects (Hamel et al., 2020, Kufner et al., 2017, Lorenz et al., 2020). The use of neuronavigation stabilizes coil positioning within an experiment and ensures consistent coil placement between sessions in real-sham within-subject designs. It has been shown to generally improve measurement conditions (Bashir et al., 2011, Julkunen et al., 2009, Sack et al., 2009). Even minor deviations in coil placement can alter MEP amplitudes (Julkunen et al., 2009, Schmidt et al., 2015). Neuronavigation is particularly recommended for longer stimulation sessions, where fatigue of the experimenter or participant may occur (Guerra et al., 2020). It is also advantageous when repositioning the coil or re-localizing the hot spot within a session, for example, when multiple measurements are interrupted by a tSMS intervention. This raises the possibility that previously reported tSMS effects may have been influenced by measurement errors due to coil displacement. However, several studies using neuronavigation have nonetheless reported inhibitory tSMS effects. For example, Davila-Pérez et al. (2019) observed inhibition with monophasic posterior-anterior TMS current direction. Although we used the same waveform, we did not observe significant effects. More recent studies (Pagge et al., 2024, 2025) likewise reported tSMS-induced modulation under neuronavigated targeting, indicating that neuronavigation alone does not preclude detecting tSMS effects. These studies, however, differ from ours in important methodological aspects – including magnet strength, cortical target, baseline excitability measurements, study design, and trial count – making direct comparison difficult. Thus, neuronavigation should be considered one methodological safeguard among several that may influence the likelihood of observing tSMS- induced changes.

Another requirement for a reliable assessment of corticospinal excitability is a minimum number of 20 pulses for single-pulse measurements and 26 stimuli for SICI (Biabani et al., 2018). Even 35 stimuli were insufficient for achieving inter-session reliability in ICF. Another study recommends at least 30 pulses to obtain stable corticospinal excitability measurements (Cuypers et al., 2014). According to Ammann et al. (2020), increasing the number of MEPs beyond approximately 20–––30 per condition yields only limited additional gains in reliability for hypothesis testing, provided that experimental conditions are well controlled, and a sufficient number of participants is included. Thus, 20 trials per condition − as used in several previous tSMS studies − can in principle be adequate for detecting group-level effects.

Nevertheless, many of the studies reporting tSMS effects applied fewer pulses per measurement, for example, 10 (Takamatsu et al., 2021) or 12 pulses (Arias et al., 2017) per condition. Such a low number of stimulation pulses calls the reliability of the measurement into question and could therefore lead to false-positive results. In our study, although we also applied only 20 stimuli for single pulses, SICI, and ICF, we recorded two such pre-intervention runs. Even in these two pre-runs, intra-session variability is clearly visible, and inter-session variability is even greater. Since none of the other tSMS studies recorded two pre-runs, the tSMS effects reported in those studies could also represent false-positive findings due to normal variability.

Another important factor, particularly regarding the variability of MEP amplitudes, is the prior activity of the target muscle. In the present study, relaxation of the stimulated hand muscle was monitored using visual and auditory feedback. Additionally, after the measurements, pulses accompanied by pre-innervation were discarded as an indication of involuntary contraction of the target muscle before stimulation. The literature repeatedly reports high variability in MEP amplitudes (e.g. Biabani et al., 2018, Kiers et al., 1993, Rossini et al., 2015, Ziemann and Siebner, 2015). Fluctuations in cortical and subcortical excitability have been suggested as a cause for high MEP variability (Kiers et al., 1993, Rossini et al., 2015). Kiers et al. (1993) postulated that increasing the level of excitability prior to stimulation could reduce MEP variability. This is similar to the proposal by Ziemann & Siebner (2015) to prime the brain through standardized protocols in order to achieve a uniform brain state before stimulation (so-called priming). According to the authors, monitoring the brain’s excitability state using EEG electrodes could also help reduce variability.

In our study, the paired-pulse paradigms also showed no modulation of inhibition or facilitation following tSMS compared to sham stimulation. Previous studies have already investigated the effect of tSMS on paired-pulse paradigms such as SICI and (S)ICF (Dileone et al., 2018, Nojima et al., 2015). Nojima et al. (2015) observed an enhancement of SICI immediately after a 20-minute tSMS intervention. Dileone et al. (2018) applied tSMS for either 10 or 30 min and found differential effects on SICI and ICF depending on the intervention duration: a 10-minute tSMS enhanced SICI and reduced ICF, whereas a 30-minute tSMS reduced SICI and enhanced ICF. These two studies suggest that a 10–20-minute tSMS may have the opposite effect on paired-pulse paradigms compared to a 30-minute application. The shorter intervention duration (10–20 min) in both studies also resulted in markedly shorter-lasting effects (0–6 min vs. 38 min). In the present study, tSMS was applied for 20 min, yet no effect on SICI or ICF was observed. However, we did demonstrate instability across multiple measurements and sessions, which was most pronounced for ICF. This variability could lead to false-positive findings regarding tSMS effects in studies that do not assess reliability.

Limitations.

The present study is the third investigation by our research group examining tSMS effects on the human motor cortex, in which no significant changes were observed. In this study, careful attention was given to a sufficiently large sample size, the use of neuronavigation, and an adequate number of stimulation pulses.

To determine the RMT, we followed the common practice of applying the intensity that elicits an MEP of over 50 µV in 5 out of 10 pulses (Groppa et al., 2012, Klomjai et al., 2015, Rossini et al., 1994). However, the reliability of this method is only 47.6% (Awiszus, 2012). When the RMT is determined so that 50 µV is exceeded in 10 out of 20 pulses, the reliability increases to 96.2%. Nonetheless, the method we used was also applied in the studies that reported a tSMS effect.

In another study, it was found that an interstimulus interval (ISI) of at least 10 s between pulses leads to more consistent MEP amplitudes than an ISI of 5 s. Shorter ISIs do not allow sufficient time for the system to return to baseline levels (Hassanzahraee et al., 2019). In the present study, the average ISI was 6.5 s. It must also be considered that cumulative single pulses can increase M1 excitability and thus lead to higher MEP amplitudes (Pellicciari et al., 2016). Consequently, increased neuronal activity from single or paired-pulse TMS could potentially have induced a modulated corticospinal excitability, possibly masking a tSMS effect with the number of pulses used in this study. Balancing the number of pulses between achieving reliable measurements and avoiding TMS-induced excitability changes remains a challenge. For this reason, ISIs were randomized in this study to prevent modulation of corticospinal excitability caused by participants’ anticipation of a pulse (Tran et al., 2021). The reliably of single pulses across measurement time points suggest that TMS-induced priming did not occur and that measurements were error-free.

Regarding the absent tSMS effect, a longer duration of tSMS intervention is suggested to enhance inhibition of the motor cortex (Dileone et al., 2018). Accordingly, a 30-minute application could have produced larger effects than the 20-minute application used in this study. However, some studies observed no change in motor cortex excitability even after 30 min of tSMS (Hamel et al., 2020, Hollis et al., 2020), while other groups reported effects with shorter stimulation durations (Oliviero et al., 2011, Silbert et al., 2013). Accordingly, the lack of effect in the present study cannot be attributed solely to the duration of magnetic application. Although Hollis et al., (2020) observed a tSMS effect on motor learning in children, they did not find any effect on motor cortex excitability, neither on single-pulse MEPs nor on paired-pulse paradigms. The authors mainly attributed this to the ongoing development of the children’s brains compared to adults. They also cited the potential influence of a concurrently performed motor task and the high interindividual variability of TMS-evoked MEPs as additional factors. Furthermore, there is a study in adults showing no significant changes in TMS-induced MEP amplitudes after a 30-minute tSMS intervention (Hamel et al., 2020).

## 5 Conclusions

Analysis of the tSMS intervention revealed no inhibitory effect on the motor cortex, regardless of the stimulated hemisphere. The increased intervention duration and participant number compared to previous studies from our group (Kufner et al., 2017, Lorenz et al., 2020), as well as the extension of the measured outcomes to include SICI and ICF, did not change the finding that tSMS produced no observable effect. Closer examination of other studies reporting significant tSMS effects indicates that these effects were observed usually in the absence of neuronavigation. Neuronavigation should be used in future studies to rule out measurement errors. For the paired-pulse paradigms, no hemispheric differences were found; however, SICI showed good reliability, while ICF showed lower reliability, consistent with previous findings. For future investigations, pre-conditioning of the motor cortex, for example via transcranial direct current stimulation, could be considered to establish a more uniform baseline state of neuronal populations, reduce MEP variability, and potentially reveal tSMS effects that may otherwise be masked.

## Author contributions

**S.L.**: Conceptualization, Formal analysis, Visualization, Writing – original draft, Writing – review & editing.

**C.H.**: Conceptualization, Data curation.

**L.B.**: Conceptualization, Data curation.

**S.G.**: Conceptualization, Formal analysis, Supervision, Resources, Writing – review & editing.

**T.K.**: Conceptualization, Formal analysis, Project administration, Supervision, Resources, Writing – original draft, Writing – review & editing.

## Funding Source

This research did not receive any specific grant from funding agencies in the public, commercial, or not-for-profit sectors.

## Declaration of competing interest

The authors declare that they have no known competing financial interests or personal relationships that could have appeared to influence the work reported in this paper.
