# Supplementary material for: Re-evaluating transcranial static magnetic stimulation (tSMS): No inhibitory effects on motor cortex across hemispheres
Source: Clin Neurophysiol Pract. 2026 Mar 4;11:163–71. doi: 10.1016/j.cnp.2026.02.008 (PMC12992518; doi:10.1016/j.cnp.2026.02.008)
Supplement: Supplementary Data 1 [file mmc1.docx]

**S1: tSMS effects on MEP amplitudes: normalized MEP**

For each participant, the two pre-intervention measurements pre 1 and pre 2 were averaged to obtain a single pre mean baseline value. All MEP amplitudes – including the two pre-intervention measurements themselves and all post-intervention time points – were then normalized by dividing each value by this individual pre mean value (pre 1/pre-mean, pre 2/pre mean…). This yielded percentage-of-baseline values for all time points and ensured that normalization was based on a stable estimate of each participant’s baseline excitability.

As in the original analysis, a repeated-measures ANOVA was conducted with the within-subject factors INTERVENTION (tSMS, sham) and TIME (pre 1, pre 2, post 1, post 2), and the between-subject factor HEMISPHERE (left, right).

The analysis revealed no significant main effects of TIME (F_(3, 108)_= 0.15, p = 0.93, η² < 0.01), INTERVENTION (F_(1,36)_ = 0.12, p = 0.73, η² < 0.01), or HEMISPHERE (F_(1,36)_ = 0.25, p = 0.62, η² < 0.01), and also no significant interactions (all p > 0.07, see Figure S1).”

**S2: Paired-pulse paradigms SICI/ICF: Normalized data**

For paired-pulse measures, SICI and ICF were normalized to the corresponding test-MEP at each time point (e.g., SICI_pre1 / TEST_pre1; ICF_pre1 / TEST_pre1).

The reliability of the normalized parameters was first assessed using correlation analyses. To avoid potential influence from the tSMS intervention, only the four *pre* values per participant were used to determine the intra- and inter-session reliability of the parameters SICI and ICF. As no hemispheric differences were observed in these analyses, results for both hemispheres are reported together for clarity. The *r*- and corresponding *p*-values from the correlation analyses are presented in Table S1.

As in the original analysis, the effects of tSMS on the paired-pulse stimulation patterns SICI and ICF were analyzed separately for each hemisphere.

Left hemisphere

The normalized MEP amplitudes were analyzed using a three-factor rmANOVA with the within-subject factors PULSE (SICI, ICF), INTERVENTION (real, sham), and TIME (pre 1, pre 2, post 1, post 2).

A statistically significant main effect was found for PULSE (F_(1,18)_ = 104.79, p ≤ 0.001, η² = 0.85).

The main effects of INTERVENTION (F_(1,18)_ = 0.82, p = 0.38, η² = 0.04) and TIME (F_(3,54)_ = 0.27, p = 0.85, η² = 0.01) were not statistically significant, nor were any of the possible interactions (all p > 0.19).

Right hemisphere

As for the left hemisphere, normalized MEP amplitudes were analyzed using a three-factor rmANOVA with the within-subject factors PULSE (SICI, ICF), INTERVENTION (real, sham), and TIME (pre 1, pre 2, post 1, post 2).

A statistically significant main effect was found for PULSE (F_(1,18)_ = 44.53, p ≤ 0.001, η² = 0.71).

The main effects of INTERVENTION (F_(1,18)_ = 1.11, p = 0.31, η² = 0.06) and TIME (F_(3,54)_ = 1.20, p = 0.32, η² = 0.06) were not statistically significant, nor were any of the possible interactions (all p > 0.17).

As for the original analyses using raw data, we analyzed the normalized SICI and ICF data separately.

For SICI, we conducted an ANOVA with the within-subject factors INTERVENTION (real, sham) and TIME (pre 1, pre 2, post 1, post 2) as well as the between-subject factor HEMISPHERE (left, right). None of the main effects (INTERVENTION: F_(1,36)_ = 1.80, p = 0.19, η² = 0.05; TIME: F_(3,108)_ = 2.16, p = 0.10, η² = 0.06; HEMISPHERE: F_(1,36)_ = 0.04, p = 0.85, η² < 0.01) or interactions (all p > 0.17) reached statistical significance (see Figure S2).

Similarly, for ICF we conducted an ANOVA with the within-subject factors INTERVENTION (real, sham) and TIME (pre 1, pre 2, post 1, post 2) and the between-subject factor HEMISPHERE (left, right). Again, none of the main effects (INTERVENTION: F_(1,36)_ = 0.68, p = 0.41, η² = 0.02; TIME: F_(3,108)_ = 0.49, p = 0.69, η² = 0.01; HEMISPHERE: F_(1,36)_ = 1.62, p = 0.21, η² = 0.04) or interactions (all p > 0.13) reached statistical significance (see Figure S2).
