# Supplementary material for: Re-evaluating transcranial static magnetic stimulation (tSMS): No inhibitory effects on motor cortex across hemispheres
Source: Clin Neurophysiol Pract. 2026 Mar 4;11:163–71. doi: 10.1016/j.cnp.2026.02.008 (PMC12992518; doi:10.1016/j.cnp.2026.02.008)
Supplement: Supplementary Data 2 [file mmc2.docx]

**Captions supplement**

**Figure S1. Effect of tSMS on normalized MEP amplitudes.**
Shown are the mean normalized MEP amplitudes for single pulses at 1 mV intensity at the different time points (pre 1, pre 2, post 1, post 2). Left panel: participants receiving stimulation over the left (dominant) hemisphere; right panel: participants receiving stimulation over the right (non-dominant) hemisphere. The intervention (20 min, grey bars) took place between pre 2 and post 1. Black = real tSMS, grey = sham stimulation. Error bars represent 95% confidence intervals.

MEP = motor-evoked potential, tSMS = transcranial static magnetic field stimulation.

**Figure S2. Effect of tSMS on SICI and ICF**

**Upper panel:** Shown are the mean MEP amplitudes for the paired-pulse paradigms SICI (left side) and ICF (right side) at the different time points (pre 1, pre 2, post 1, post 2) during real tSMS (black) and sham (grey) stimulation. The intervention (20 min, grey bar) took place between pre 2 and post 1. Error bars represent 95% confidence intervals.

**Lower panel:** Shown are the mean normalized MEP amplitudes for the paired-pulse paradigms SICI (left side) and ICF (right side) at the different time points (pre 1, pre 2, post 1, post 2) during real tSMS (black) and sham (grey) stimulation. The intervention (20 min, grey bar) took place between pre 2 and post 1. Error bars represent 95% confidence intervals.

MEP = motor-evoked potential; tSMS = transcranial static magnetic field stimulation; SICI = short-interval cortical inhibition; ICF = intracortical facilitation.
