# Supplementary figures and images for: Re-evaluating transcranial static magnetic stimulation (tSMS): No inhibitory effects on motor cortex across hemispheres
Source: Clin Neurophysiol Pract. 2026 Mar 4;11:163–71. doi: 10.1016/j.cnp.2026.02.008 (PMC12992518; doi:10.1016/j.cnp.2026.02.008)

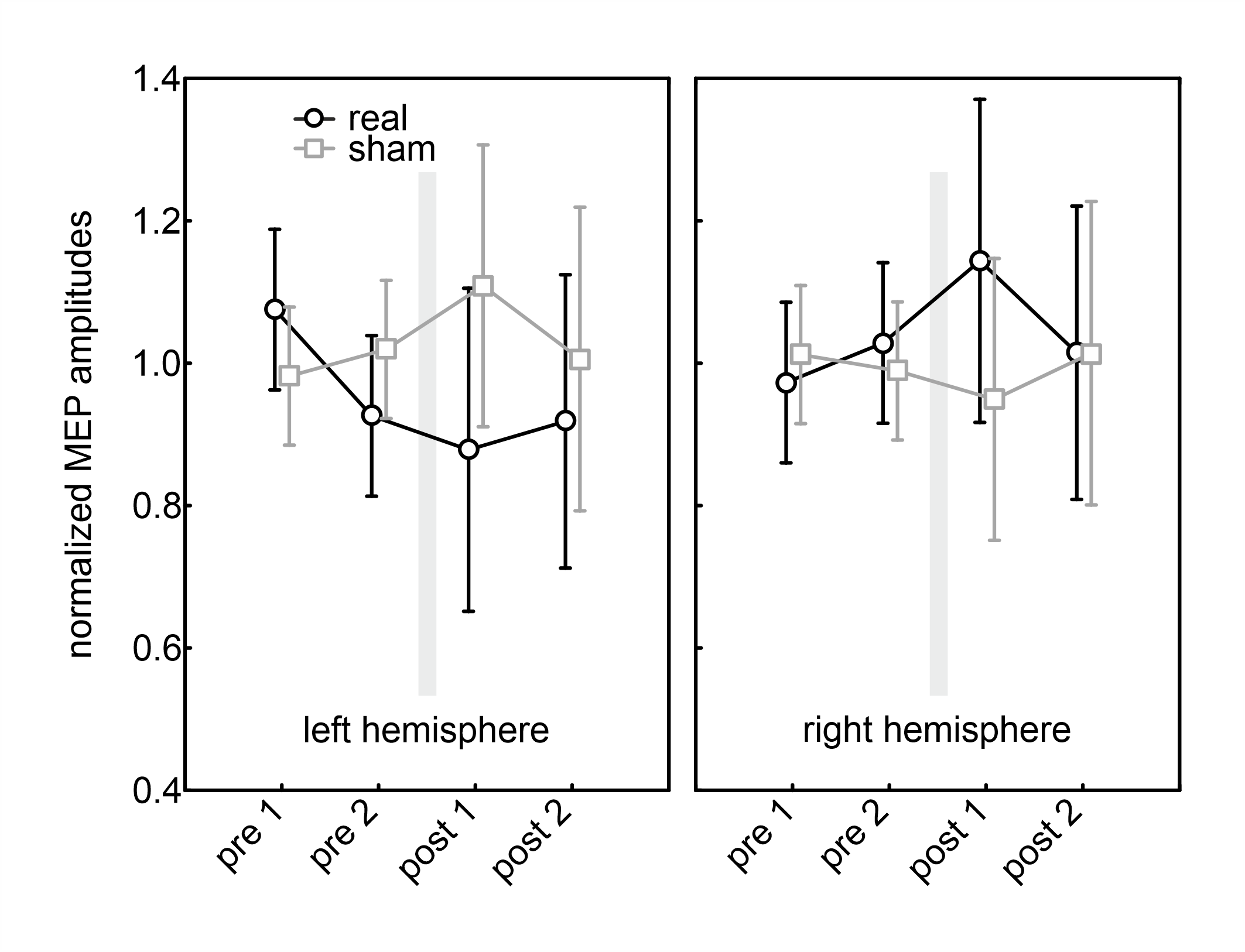

Supplement: Supplementary Data 3 [file mmc3.zip › Supplement_Figure_S1.tiff]

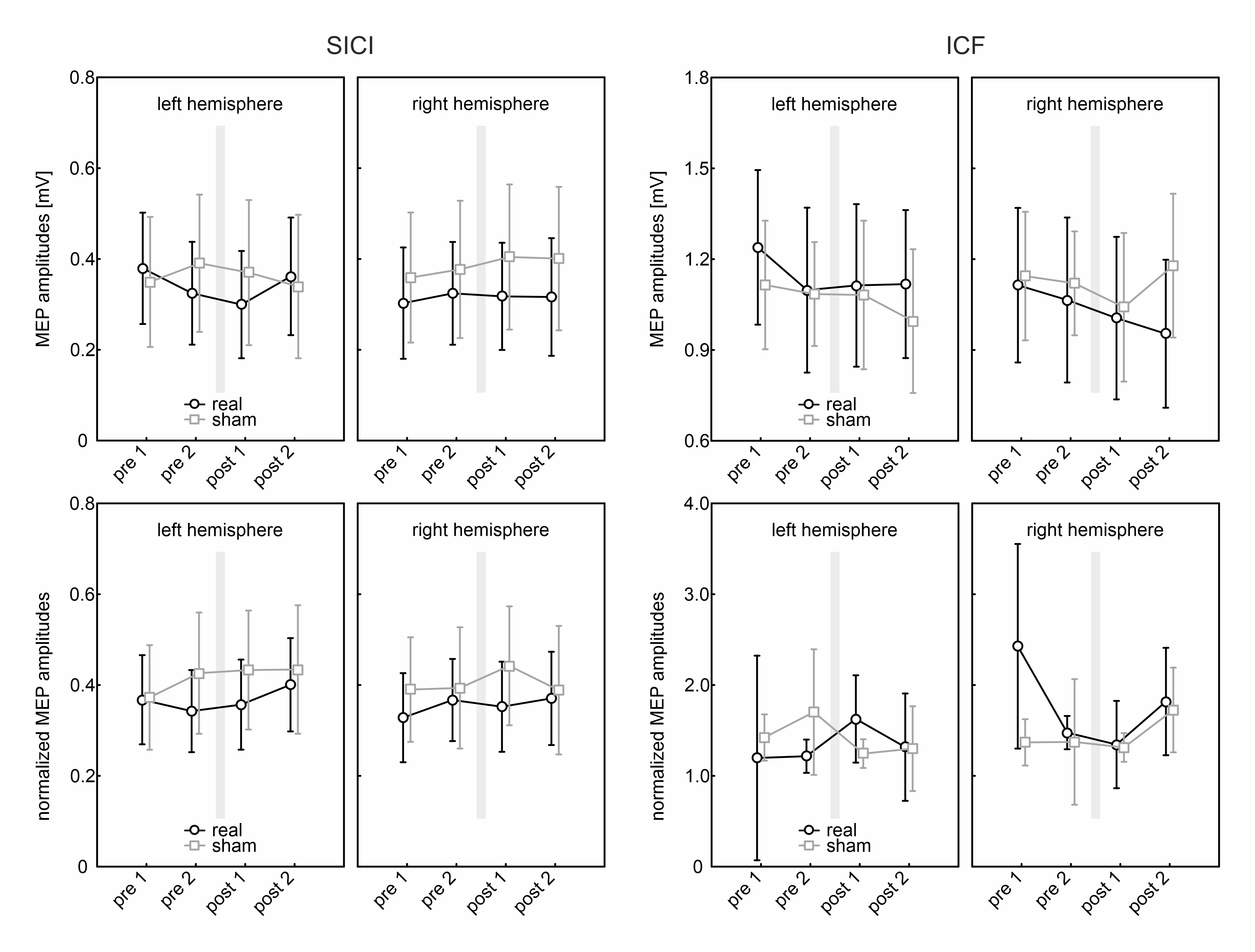

Supplement: Supplementary Data 4 [file mmc4.zip › Supplement_Figure_S2.tiff]
