# Supplementary material for: Re-evaluating transcranial static magnetic stimulation (tSMS): No inhibitory effects on motor cortex across hemispheres
Source: Clin Neurophysiol Pract. 2026 Mar 4;11:163–71. doi: 10.1016/j.cnp.2026.02.008 (PMC12992518; doi:10.1016/j.cnp.2026.02.008)
Supplement: Supplementary Data 5 [file mmc5.docx]

**Supplementary tables**

|  |  | **SICI** | **ICF** |
| --- | --- | --- | --- |
| **intra- session** | s1 pre1/s1 pre2 | r = 0.86 p < 0.01 | r = 0.06 p = 0.72 |
|  | s2 pre1/s2 pre2 | r = 0.86 p < 0.01 | r = 0.48 p < 0.01 |
| **inter- session** | s1 pre1/s2 pre1 | r = 0.50 p < 0.01 | r < 0.01 p = 0.98 |
|  | s1 pre1/s2 pre2 | r = 0.50 p < 0.01 | r = 0.11 p = 0.50 |
|  | s1 pre2/s2 pre1 | r = 0.54 p < 0.01 | r = 0.05 p = 0.76 |
|  | s1 pre2/s2 pre2 | r = 0.56 p < 0.01 | r = 0.20 p = 0.24 |

**Table S1. Correlation analyses.**Shown are the *r*- and *p*-values for the correlations of the mean normalized MEP amplitudes for the parameters SICI and ICF across the four measurement time points: s1 pre1 (session 1, measurement 1), s1 pre2 (session 1, measurement 2), s2 pre1 (session 2, measurement 1), and s2 pre2 (session 2, measurement 2).
